# Gastrointestinal microbiota profile and clinical correlations in advanced *EGFR*-WT and *EGFR*-mutant non-small cell lung cancer

**DOI:** 10.1186/s12885-022-10050-3

**Published:** 2022-09-08

**Authors:** Woraseth Saifon, Insee Sensorn, Narumol Trachu, Songporn Oranratnachai, Angkana Charoenyingwattana, Chakkaphan Runcharoen, Nanamon Monnamo, Warawut Sukkasem, Pimpin Inchareon, Thitiporn Suwatanapongched, Phichai Chansriwong, Touch Ativitavas, Ravat Panvichian, Wasun Chantratita, Thanyanan Reungwetwattana

**Affiliations:** 1 Division of Medical Oncology, Department of Medicine, Faculty of Medicine Ramathibodi Hospital, Mahidol University, Bangkok, Thailand; 2 Department of Medicine, Golden Jubilee Medical Center, Nakorn Pathom, Thailand; 3 Center for Medical Genomics, Faculty of Medicine Ramathibodi Hospital, Mahidol University, Bangkok, Thailand; 4 Research Center, Faculty of Medicine Ramathibodi Hospital, Mahidol University, Bangkok, Thailand; 5 Sriphat Medical Center, Faculty of Medicine, Oncology Clinic, Chiang Mai University, Chiang Mai, Thailand; 6 Department of Diagnostic and Therapeutic Radiology, Faculty of Medicine Ramathibodi Hospital, Mahidol University, Bangkok, Thailand; 7 Ramathibodi Lung Cancer Consortium, Faculty of Medicine Ramathibodi Hospital, Mahidol University, Bangkok, Thailand; 8 Department of Pathology, Faculty of Medicine Ramathibodi Hospital, Mahidol University, Bangkok, Thailand

**Keywords:** Microbiota, Microbiome, *EGFR*-mutant, NSCLC, Lung cancer

## Abstract

**Introduction:**

Difference in clinical responses to cancer therapy in each patient is from several factors. Gastrointestinal microbiota is one of the reasons. However, this correlation remains unknown. This study aims to explore correlation between gastrointestinal microbiota profile and clinical outcomes in Thai advanced non-small cell lung cancer (NSCLC) according to epidermal growth factor receptor (*EGFR*) status.

**Methods:**

We enrolled 13 patients with advanced *EGFR*–wild-type (WT) NSCLC who received chemotherapy and 15 patients with *EGFR*-mutant NSCLC who received *EGFR* tyrosine kinase inhibitors. We collected fecal samples at baseline and first disease evaluation and performed 16S rRNA gene sequencing by NGS to assess microbiota profile. The correlations between gastrointestinal microbiota and clinical variables were studied.

**Results:**

The clinical characteristics were balanced between the cohorts, excluding significantly higher albumin levels in the *EGFR*-mutant group. Albumin was the only significant clinical factor affecting the treatment response in multivariate analysis (ORR 15.6%, *P* = 0.03). Proteobacteria counts were higher in the *EGFR*-WT group, whereas Bacteroidetes and Firmicutes counts were higher in the *EGFR*-mutant group. The alpha diversity of the gastrointestinal microbiome was significantly higher in the *EGFR*-mutant group (Shannon index: 3.82 vs. 3.25, *P* = 0.022). Following treatment, Proteobacteria counts were lower and Bacteroidetes and Firmicutes counts were higher in both cohorts; the changes were more prominent in the *EGFR*-WT cohort. No significant correlation between microbiota profile and treatment response were demonstrated in our study. However, beta diversity was significantly different according to severity of adverse events. Enrichment of Clostridia and Bacteroidia was associated with higher adverse event risk in the *EGFR*-WT cohort.

**Conclusions:**

Proteobacteria was dominant in Thai lung cancer patients both *EGFR*-WT and *EGFR*-mutant, and this phylum maybe associate with lung cancer carcinogenesis. Chemotherapy altered the gastrointestinal microbiota, whereas *EGFR*-TKIs had less effects. Our findings highlight the potential predictive utility of the gastrointestinal microbiota for lung cancer carcinogenesis. Studies with larger cohorts and comparison with the healthy Thai population are ongoing to validate this pilot study.

**Supplementary Information:**

The online version contains supplementary material available at 10.1186/s12885-022-10050-3.

## Introduction

Non-small cell lung cancer (NSCLC) accounts for approximately 80–85% of all lung cancers worldwide [1]. In Thailand, NSCLC is the most common cancer and the second most common cause of cancer-related death after biliary tract cancer [2, 3]. Although advancements in NSCLC treatment have prolonged survival, platinum-based doublet chemotherapy, which is a mainstay of treatment for patients with advanced NSCLC in developing countries [4], carries an overall response rate (ORR) of approximately 30–40% and overall survival (OS) of 8–12 months [5]. Furthermore, Asian patients with lung adenocarcinoma have a high prevalence of epidermal growth factor receptor (*EGFR*) mutation (50%) [6]. In tyrosine kinase inhibitor (TKI)-sensitive *EGFR*-mutant NSCLC, EGFR-TKIs significantly improve survival and ORRs (60–70%) [7, 8]. However, some patients with TKI-sensitive *EGFR*-mutant NSCLC do not respond to EGFR-TKIs. In addition, patients with *EGFR*exon 19 deletion appear to have a longer duration of response and higher ORRs [7, 8]. To date, no predictive biomarkers of the efficacy and toxicity of chemotherapy and EGFR-TKIs in the treatment of NSCLC have been identified.

The human microbiota, which consists of more than 1000 species, approximately 97% of which reside in the intestine, is a key focus of global research [9]. Research on bacterial 16S ribosomal RNA using metagenomics technology including next-generation sequencing (NGS) has provided several insights into the human microbiota. The gastrointestinal microbiota can affect host immune responses in various ways. Previous research reported that the gastrointestinal microbiota plays an important role in drug efficacy and toxicity in response to therapy [10–14]. Conversely, dysbiosis can affect patient outcomes. Several factors can alter the microbiome composition such as medications, diet, prebiotics, probiotics [15–21], and cancer therapies. Several studies found that chemotherapies, such as oxaliplatin and cyclophosphamide, can directly cause epithelial damage. Translocation of gastrointestinal microbes and their biologic products across the gastrointestinal epithelium can stimulate T cell expansion in lymphoid tissue [9, 22, 23] and result in different immune responses that might affect cancer. Inhibition of *EGFR *in the gastrointestinal tract leads to changes in intestinal architecture, thereby resulting in microbiota changes by increasing in bowel movement frequency [24]. Understanding the interactions among the gastrointestinal microbiota, therapies, and cancer response is necessary for the development of microbiota-targeting approaches to improve treatment efficacy.

Most previous studies [12–14] in various tumor types involved patients who received immunotherapy. In these studies, certain microbes and high microbial diversity were related to different clinical outcomes. There is limited knowledge regarding the gastrointestinal microbiota profile of patients with advanced *EGFR*-WT and *EGFR*-mutant lung cancer who received chemotherapy and targeted therapy.

This study explored the gastrointestinal microbiota profiles of patients with advanced *EGFR*-WT and *EGFR*-mutant NSCLC who received chemotherapy and EGFR-TKIs, respectively. We analyzed gastrointestinal microbiota profiles before and after treatment initiation and their associations with clinical features, especially treatment outcomes.

## Materials and methods

### Patients and study design

This prospective study enrolled treatment-naïve patients with stage III–IV *EGFR*-WT or *EGFR*-mutant NSCLC in Ramathibodi Hospital, Mahidol University (Bangkok, Thailand) between June 2020 and January 2021. The eligibility criteria were an age of at least 18 years and Eastern Cooperative Oncology Group performance status (ECOG PS) of 0–2. The diagnosis of NSCLC was on the basis of histopathological analysis, and stage was classified according to the TNM classification (AJCC 8^th^ edition). The *EGFR* status was confirmed in all patients using tissue samples by real-time PCR (Therascreen, Qiagen). Patients underwent stool collection after receiving an explanation of the process. All participants had measurable disease according to computed tomography or magnetic resonance imaging using Response Evaluation Criteria in Solid Tumors version 1.1 (RECIST 1.1). This study and all experimental protocols were approved by The Human Research Ethics Committee of Ramathibodi Hospital, Mahidol University, Bangkok, Thailand with the IRB number of COA. MURA2021/305. All methods were carried out in accordance with relevant guidelines and local regulations. Informed consent was obtained from all patients in this study.

Patients were classified into two cohorts: the *EGFR*-WT cohort, in which patients were treated with standard platinum-based doublet chemotherapy regimens every 3 weeks for 4–6 cycles or until unacceptable toxicity; and the *EGFR*-mutant cohort, in which patients received any-generation *EGFR*-TKIs until progression or until unacceptable toxicity. The response to treatment was evaluated 8–12 weeks after treatment initiation. In this study, patients were categorized as responders and non-responders. In the *EGFR*-WT cohort, responders comprised patients with complete responses (CRs), partial responses (PRs), and stable disease (SD), and patients with progressive disease (PD) were classified as non-responders. In the *EGFR*-mutant cohorts, responders included patients with CR or PR, whereas those with SD or PD were classified as non-responders.

### Procedures

Informed consent was received from all eligible patients. Paired fecal samples were collected from each patient at baseline (before treatment initiation) and at the first disease evaluation (2–3 months after treatment initiation). The proper size of fecal samples was a grain of rice (50–100 mg/time point), and samples were collected using StoolFiX™ (Isohelix, Cell Projects) containing 500 μL of lysis buffer and 25 μL of proteinase K and stored at room temperature until DNA extraction.

Clinical data (age, sex, underlying disease, smoking status, performance status, body weight, body mass index, type of lung cancer, type of *EGFR* mutation, metastatic site, laboratory results, and history of gastrointestinal tract surgery) were collected at baseline by reviewing the Electronic Medical Record (EMR). We also interviewed the patients in person for the history of antibiotics, proton pump inhibitors, prebiotics, or probiotics usage within 1 month prior starting the treatment. During the treatment, we collected the response and adverse events (AEs) of treatment of each patient. AEs were assessed and graded according to the Common Terminology Criteria for Adverse Events (CTCAE) version 4. CTCAE grading ≥ 2 AEs were categorized as severe AEs, and CTCAE grading 0–1 were classified as mild AEs.

### Bacterial fecal DNA extraction

Bacterial fecal DNA extraction was performed at the Center for Medical Genomics, Faculty of Medicine, Ramathibodi Hospital, Mahidol University. The fecal sample was mixed with 20 μL of PK solution and incubated in a 60◦C water bath for 1 h. Then, the sample tubes were centrifuged at 12,000 × *g* for 10 min. The supernatant was removed, and then the tubes were briefly re-spun to remove any remaining supernatant. Next, 100 μL of elution buffer were added. The subsequent extraction procedure was performed according to the manufacturer’s instruction. Metagenomic DNA was extracted using a QIAamp PowerFecal DNA Kit (QIAGEN, Germany). DNA quality and quantity were assessed using a NanoDrop One spectrophotometer (Thermo Fisher Scientific, USA) and Qubit® 3.0 fluorometer (Thermo Fisher Scientific).

### Polymerase chain reaction (PCR) and sequencing

To determine the bacterial composition in feces, construction of an Illumina metagenomic sequencing library was performed via two-step PCR. The first PCR was performed to amplify the V3–V4 hypervariable region of the bacterial 16S rRNA gene using the primers 338F (5′-ACTCCTACGGGAGGCAGCAG-3′) and 806R (5′-GGACTACHVGGGTWTCTAAT-3′). The second PCR was performed to add the index sequences for the Illumina sequencer with the barcode sequence using a Nextera XT Index kit (Illumina, San Diego, CA, USA). The prepared libraries were subjected to sequencing of the paired-end 2 × 250 bases using a MiSeq Reagent Kit v2 on the MiSeq (Illumina).

### Bioinformatics analysis

Raw sequencing data were cleaned using FLASH software and analyzed using Quantitative Insights into Microbial Ecology 2 (QIIME 2) [25]. Demultiplexed cleaned sequence data were denoised using DADA2, and then the amplicon sequence variant (ASVs) counts per sample was generated and grouped by different taxonomic levels (phylum, class, order, family, genus, species). Taxonomic assignment was based on the SILVA 138 ribosomal (rRNA) database.

The diversity within the gastrointestinal community was assessed via alpha and beta diversity analyses. To assess alpha diversity, Shannon’s index, which reflects the complexity of species diversity within samples, was calculated. To assess beta diversity, principal coordinate analysis (PCoA) was performed using the Bray–Curtis index to compare community dissimilarity. Linear discriminant analysis with effect size (LEfSe) was used to compared our groups of interest to identify biomarkers and visualize the result using a taxonomic bar chart [26].

### Statistical analysis

The patients’ baseline characteristics were reported using descriptive statistics. Comparisons between the *EGFR*-WT and *EGFR*-mutant cohorts were performed using the Chi-squared or Fisher’s exact test for categorical variables and Student’s *t*-test or the Mann–Whitney U test for continuous variables as appropriate. Progression-free survival (PFS) was estimated using the Kaplan–Meier method as the time from diagnosis to disease progression or death from any cause. The associations of clinical variables with alpha diversity were explored using univariate and multivariate linear regression analysis. The associations of clinical variables including alpha diversity with the response to treatment were analyzed using univariate and multivariate logistic regression analysis and presented as odds ratios (ORs) with 95% confidence intervals (CIs) and P-values. All statistical analyses were performed using Stata version 16. *P* < 0.05 indicated statistical significance.

## Results

### Patient characteristics and clinical outcomes

From June 2020 to January 2021, 42 patients were screened. Twenty-eight patients (13 with *EGFR*-WT NSCLC and 15 with *EGFR*-mutant NSCLC) met the study’s criteria and finally included in this analysis. (Supplemental Fig. 1 ).

**Fig. 1:**
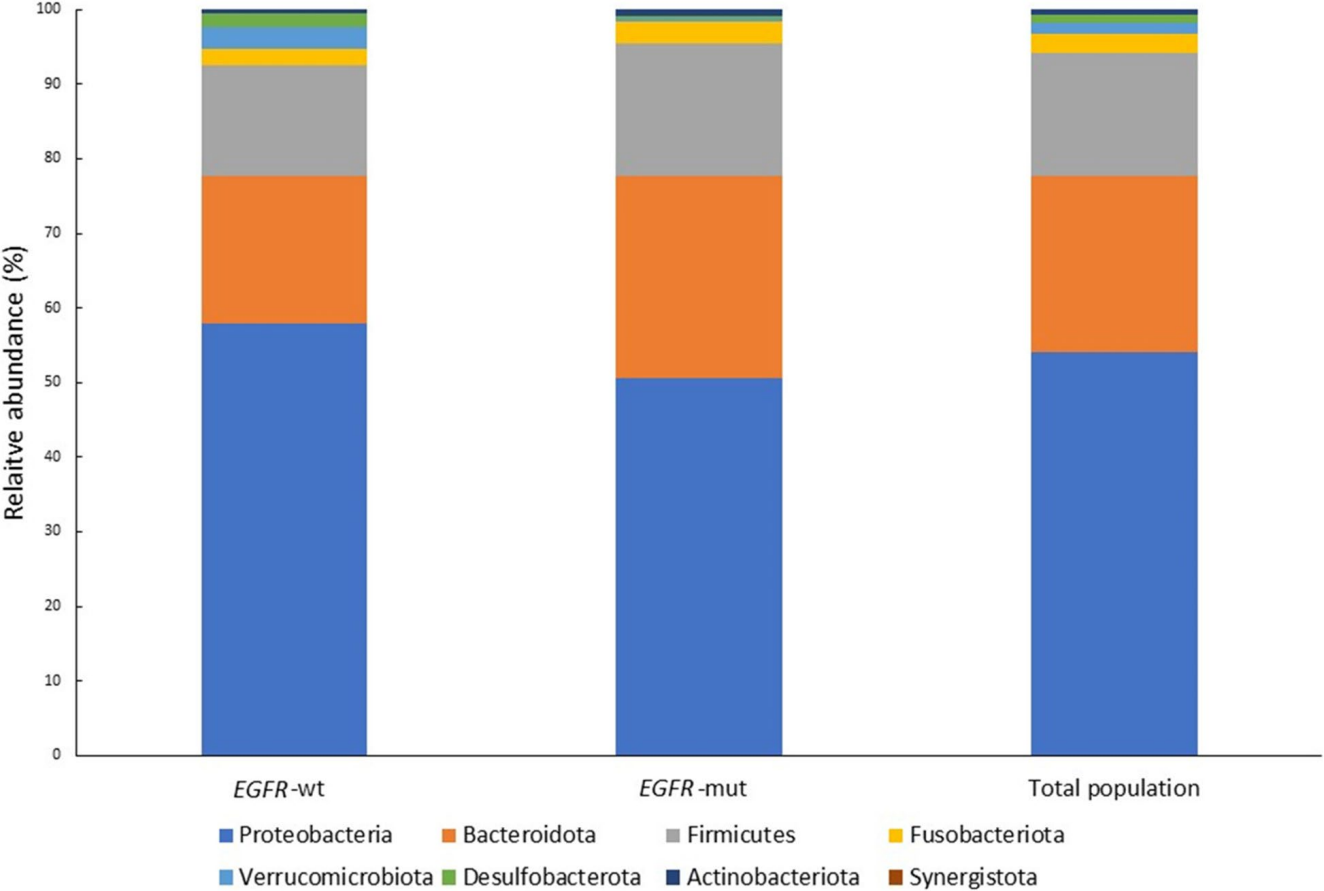
Relative abundance of gastrointestinal microbiota phyla at baseline in the *EGFR*-WT and *EGFR*-mutant cohorts. *EGFR*-wt*:* epidermal growth factor receptor wild-type; *EGFR*-mut: epidermal growth factor receptor mutant

In the *EGFR*-WT cohort, the median patient age was 63 years. Most patients were women (69.2%) and never-smokers (76.9%). Nearly half of the patients had normal BMI (46.1%), and 92.2% had an ECOG PS of 0–1 (Table 1). Hypertension (46.1%) was the most common underlying disease, followed by diabetes mellitus (17.8%). Adenocarcinoma was the most common histopathologic subtype (84.6%), whereas two patients had squamous histopathology. All patients received carboplatin/paclitaxel as the first-line chemotherapy.

**Table 1:** Baseline clinical characteristics, choice of treatment, and adverse events of patients with *EGFR*-WT and *EGFR*-mutant NSCLC

Variable	Total population (*N* = 28)	*EGFR*-WT (*N* = 13)	*EGFR*-mutant (*N* = 15)	*P*
Sex, no. (%)				0.13
• Male	14 (50)	4 (30.7)	10 (66.6)	
• Female	14 (50)	9 (69.2)	5 (33.3)	
Age (years)				0.77
• Mean ± SD	63.6 ± 8.8	63.0 ± 9.7	64.0 ± 8.2	
BW (kg)				0.28
• Median	50.3	50.7	50	
• Range	42—76	45—76	(42—76)	
BMI (kg/m^2^), no. (%)				0.43
• Underweight (<18.5)	3 (10.7)	0 (0)	3 (20.0)	
• Normal (18.5–22.9)	13 (46.4)	6 (46.1)	7 (46.6)	
• Overweight (23–24.9)	6 (21.4)	4 (30.7)	2 (13.3)	
• Obese (> 25)	6 (21.4)	3 (23.0)	3 (20.0)	
WHO performance status (ECOG), no. (%)				0.69
• 0	7 (25.0)	2 (15.3)	5 (33.3)	
• 1	19 (67.8)	10 (76.9)	9 (60.0)	
• 2	2 (7.1)	1 (7.6)	1 (6.6)	
Smoking status, no. (%)				0.48
• Never-smoker	20 (71.4)	10 (76.9)	10 (66.6)	
• Ex-smoker	5 (17.8)	1 (7.6)	4 (26.6)	
• Current smoker	3 (10.7)	2 (15.3)	1 (6.67)	
Underlying disease, no. (%)				
• Diabetes mellitus	5 (17.8)	3 (23.0)	2 (13.3)	0.64
• Hypertension	12 (42.8)	6 (46.1)	6 (40.0)	0.74
• Dyslipidemia	7 (25.0)	1 (7.6)	6 (40.0)	0.08
• Chronic obstructive pulmonary disease	0 (0)	0 (0)	0 (0)	
• Inflammatory bowel disease	0 (0)	0 (0)	0 (0)	
Previous GI tract surgery, no. (%)	1 (3.5)	0 (0)	1 (6.6)	> 0.99
Drug use in past 4 weeks, no. (%)				
• Antibiotics	3 (10.7)	2 (15.3)	1 (6.6)	0.58
• Proton pump inhibitors	5 (17.8)	1 (7.6)	4 (26.6)	0.33
• Laxatives	8 (28.5)	4 (30.7)	4 (26.6)	> 0.99
• Prebiotics/probiotics	8 (28.5)	3 (23.0)	5 (33.3)	0.69
• Supplement	4 (14.2)	2 (15.3)	2 (13.3)	0.78
Non-small cell lung cancer Histologic subtype, no. (%)				0.21
• Adenocarcinoma	25 (85.2)	11 (84.6)	14 (93.3)	
• Squamous cell carcinoma	2 (7.1)	2 (15.3)	0 (0)	
• Adenosquamous cell carcinoma	1 (3.5)	0 (0)	1 (6.67)	
Stage at diagnosis, no. (%)				0.82
• IIIb	1 (3.5)	0 (0)	1 (6.6)	
• IIIc	2 (7.1)	1 (7.6)	1 (6.6)	
• IVa	19 (67.8)	10 (76.9)	9 (60.0)	
• IVb	6 (21.4)	2 (15.3)	4 (26.6)	
Albumin (g/dL), no. (%)				0.02
< 3.2 ≥ 3.2	12 (42.8) 16 (57.1)	9 (69.2) 4 (30.7)	3 (20.0) 12 (80)	
*EGFR* status, no. (%)				
• Wild-type		13 (100)	0 (0)	
• Exon 19 deletion			8 (53.3)	
• Exon 21 L858R			5 (33.3)	
• Uncommon mutations (G719X, S768I, L861Q)			2 (13.3)	
Treatment regimen, no. (%)				
• Carboplatin/paclitaxel		13 (100)		
• Gefitinib			4 (26.6)	
• Erlotinib			4 (26.6)	
• Osimertinib			2 (13.3)	
• Others			5 (33.3)	
Adverse events, no. (%)				0.66
Grade< 2 Grade≥ 2	12 (42.8) 16 (57.1)	5 (38.4) 8 (61.5)	7 (46.6) 8 (53.3)	

*BMI* Body mass index, *BW* Body weight, *EGFR* Epidermal growth factor receptor, *GI* Gastrointestinal, *WHO* World Health Organization

In the *EGFR*-mutant cohort, the median patient age was 64 years. Most patients were men (66.6%) and never-smokers (66.6%). Nearly half of the patients had normal BMI (46.6%), and 14 (93.3%) patients had an ECOG PS of 0–1. Most patients had adenocarcinoma (93.3%), and one patient had adenosquamous histology. Hypertension (40.0%) was the most common underlying disease, followed by dyslipidemia (40.0%). Exon 19 deletion and exon 21 L858R mutations were the most common *EGFR* mutations in this population (86.6%). The remaining patients had uncommon *EGFR* mutations. Half of the patients (53.2%) received first-generation *EGFR*-TKIs, whereas 13.3% received third-generation *EGFR*-TKIs.

The most common metastatic sites in both cohorts were the pleura and lungs. The *EGFR*-mutant cohort had a significantly better nutritional status (albumin ≥ 3.2 g/dL) than the *EGFR*-WT cohort (80.0% vs. 30.7%, *P* = 0.02). Other clinical factors were similar between the two cohorts (Table 1).

The response to treatment in the *EGFR*-WT cohort was as follows: PR, 7.6%; SD, 38.4%; and PD, 53.8%. Meanwhile, the response in the *EGFR*-mutant cohort was as follows: PR, 66.6%; SD, 26.6%; and PD, 6.67% Thus, the response rates in the *EGFR*-WT and *EGFR*-mutant cohorts were 7.6 and 66.6%, respectively (Supplemental Table 1). Furthermore, median PFS was 3.5 months in the *EGFR*-WT cohort, versus 7.9 months in the *EGFR*-mutant cohort.

Albumin was significantly predictive of the response of treatment in both univariate and multivariate analyses. Patients with albumin ≥ 3.2 g/dL were significantly more likely to respond to treatment than those with albumin < 3.2 g/dL (ORR = 15.7, 95% CI = 1.3–182, *P* = 0.028, Table 2). In univariate analysis, patients with *EGFR*-mutant NSCLC were also more likely to respond to treatment than those with *EGFR*-WT NSCLC (ORR = 24.0, 95% CI = 2.4–240.6, *P* = 0.0007), whereas the small size of the *EGFR*-WT cohort precluded multivariate analysis.

**Table 2:** Univariate and multivariate logistic regression analyses of treatment response (based on the overall response rate) and clinical characteristics in the total population (*N* = 28)

Clinical characteristics		Response/total, (N)	Univariate analysis		Multivariate analysis	
			**Odds ratio** **(95% CI)**	***P***	**Odds ratio** **(95% CI)**	***P***
**Sex**	Male	6/14	ref			
	Female	5/14	0.7 (0.2, 3.4)	0.70		
**BMI**	Normal/underweight	7/16	ref			
	Overweight/obese	4/12	0.6 (0.1, 3.0)	0.58	0.8 (0.1, 6.1)	0.84
**Smoking status**	Never-smoker	7/20	ref			
	Ever-smoker	4/8	1.9 (0.4, 9.8)	0.47		
**Antibiotics**	Yes	1/3	0.8 (0.1, 9.4)	0.82		
	No	10/25	ref			
**PPIs**	Yes	3/5	2.8 (0.4, 20.5)	0.31		
	No	8/23	ref			
**Laxatives**	Yes	2/8	0.4 (0.1, 2.5)	0.34		
	No	9/20	ref			
**Prebiotics/Prebiotics**	Yes	4/8	1.9 (0.4, 9.8)	0.47	1.8 (0.2, 14.2)	0.58
	No	7/20	ref			
**Supplement**	Yes	3/4	5.6 (0.5, 63.3)	0.16		
	No	12/24	ref			
**Lung/pleural metastasis**	Yes	5/17	0.3 (0.1, 1.7)	0.19	0.8 (0.1, 6.3)	0.84
	No	6/11	ref			
**Brain metastasis**	Yes	3/5	2.8 (0.4, 20.4)	0.31	1.5 (0.1, 18.1)	0.75
	No	8/23	ref			
**Albumin**	< 3.2 g/dL	1/12	ref			
	≥ 3.2 g/dL	10/16	18.3 (1.9, 179.9)	0.01	15.6 (1.3, 182.0)	0.03
**Cohort**	*EGFR*-WT	1/13	ref			
	*EGFR-*mutant	10/15	23.9 (2.4, 240.6)	0.0007	not available	
**Shannon diversity index**		11/28	1.2 (0.4, 3.8)	0.77	1.0 (0.3, 4.2)	0.97

*BMI* Body mass index, *EGFR* Epidermal growth factor receptor, *PPI* Proton pump inhibitor, *WT* Wild-type

Most patients in the *EGFR*-WT cohort (61.5%) experienced severe AEs (Common Terminology Criteria for Adverse Events: CTCAE ≥ 2), and 53.3% of patients in the *EGFR*-mutant cohort experienced all grades AEs. The most common AEs in the *EGFR*-WT cohort were anemia (92.3%) and vomiting (25.0%), whereas those in the *EGFR*-mutant cohort were rash (60.0%), anemia (53.0%), and diarrhea (20%). All patients in EGFR-mutant cohort developed mild degree of adverse events (CTCAE < 2), there was no severe AEs. There were no significant differences in antibiotic, proton pump inhibitor, and laxative use between the cohorts.

### Gastrointestinal microbiota in patients with lung cancer at baseline

In the entire population, the five most abundant bacteria at the phylum level at baseline were Proteobacteria (53.9%), Bacteroidetes (23.7%), Firmicutes (16.4%), Fusobacteriota (2.5%), and Verrucomicrobia (1.5%) (Fig. 1). The relative abundance of these bacteria was similar between the groups, but Proteobacteria counts were higher in the *EGFR*-WT cohort whereas Bacteroidetes and Firmicutes counts were higher in the *EGFR*-mutant cohort, albeit without significance.

### Compositional differences of the gastrointestinal microbiota according to the response to treatment

**Fig. 2:**
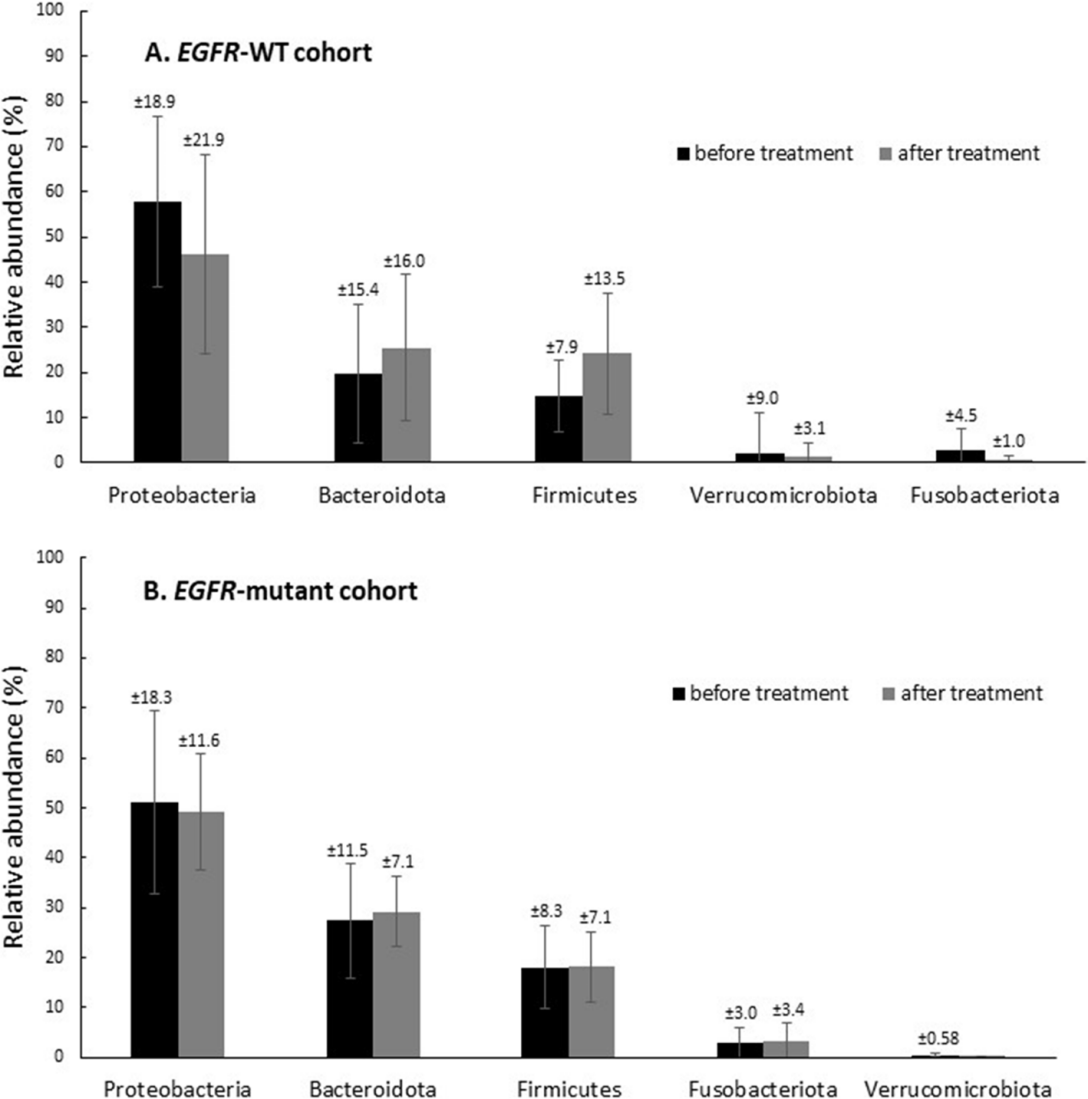
Comparison of relative abundance of gut microbiota phyla before treatment (T1) and after treatment (T2). A. *EGFR*-WT cohort B. *EGFR*-mutant cohort

We found some alterations in the microbiota composition between before and after treatment. After patients with *EGFR*-WT NSCLC received chemotherapy, the relative abundance of Proteobacteria decreased (46.2%), whereas that of Bacteroidetes (25.5%) and Firmicutes (24.1%) increased. There was no distinct change in the microbiota composition in the *EGFR*-mutant cohort (Fig. 2).

To investigate whether the microbiota composition was associated with the response to chemotherapy or *EGFR*-TKIs, we descriptively compared the relative abundance of gastrointestinal bacteria at the phylum level between responders and non-responders. The results revealed no significant differences between responders and non-responders in both cohorts (Supplement Fig. 2A and B). However, we observed that Desulfobacterota was enriched (21.1%) among partial responders in the *EGFR*-WT cohort, whereas Actinobacteria was enriched in patients with PD in the *EGFR-*mutant cohort (Supplemental Fig. 3A and B).

**Fig. 3:**
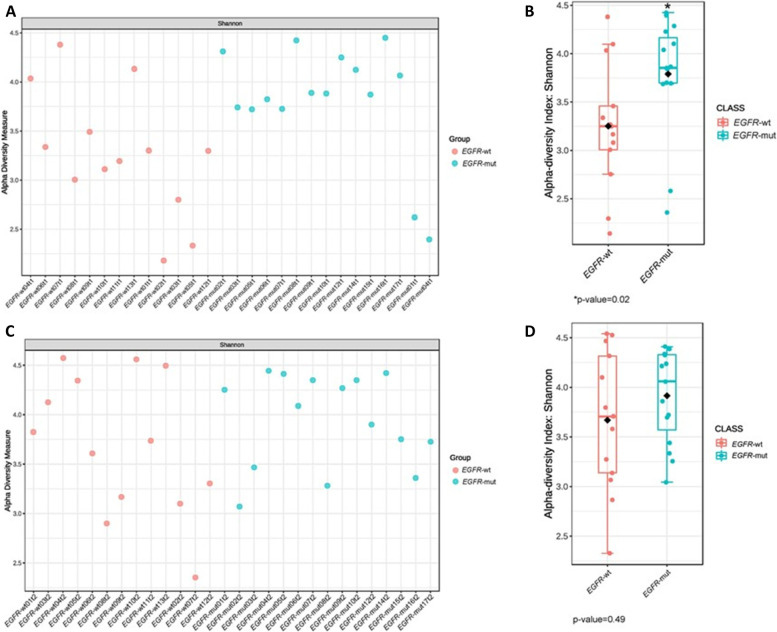
Comparison of alpha diversity in EGFR-WT and EGFR-mutant cohorts. A Shannon index in every patient at baseline B. Shannon index between 2 cohorts at baseline. B Shannon index in every patient after treatment D. Shannon index between 2 cohorts after treatment. *EGFR*-wt*:* epidermal growth factor receptor wild-type; *EGFR*-mut: epidermal growth factor receptor mutant

### Differences of gastrointestinal microbiota diversity

Shannon index (alpha diversity) was calculated to show species abundance in this population. The results revealed that the alpha diversity of the gastrointestinal microbiota was significantly higher in the *EGFR-*mutant cohort than that in the *EGFR*-WT cohort at baseline (3.85 vs. 3.25, *P* = 0.022, Fig. 3). Consistent with the results of multivariate analysis, the *EGFR* mutation status was significantly associated with the Shannon index (Table 3). Univariate linear regression analysis between clinical factors and the Shannon index revealed that gender and the *EGFR* mutation status were significantly associated with the Shannon index. Specifically, women had a lower Shannon index than men (3.21 ± 0.63 vs. 3.86 ± 0.55, *P* = 0.008), whereas patients with *EGFR*-mutant NSCLC had a higher Shannon index than those with *EGFR*-WT NSCLC. After adjustment for potential confounding factors in multivariate linear regression analysis, only the *EGFR* status remained significantly associated with the Shannon index (3.79 ± 0.59 vs. 3.25 ± 0.65, *P* = 0.047).

**Table 3:** Univariate and multivariate linear regression analyses of the pre-treatment Shannon diversity index and clinical characteristics in the total population (*N* = 28)

**Factors**		**N**	**Shannon diversity**	**Univariate analysis**		**Multivariate analysis**	
				**Coefficient** **(95% CI)**	***P***	**Coefficient** **(95% CI)**	***P***
**All patients**		28	3.5 ± 0.7				
**Sex**	Male	14	3.9 ± 0.6	ref			
	Female	14	3.2 ± 0.6	-0.6 (-1.1, -0.2)	0.01	-0.8 (-1.6, 0.1)	0.08
**BMI**	Normal/underweight	16	3.5 ± 0.7	ref			
	Overweight/obese	12	3.5 ± 0.7	-0.1 (-0.5, 0.5)	0.96	0.5 (-0.3, 1.2)	0.24
**Smoking status**	Never-smoker	20	3.5 ± 0.7	ref			
	Ever-smoker	8	3.7 ± 0.7	0.3 (-0.3, 0.9)	0.33	-0.8 (-2.0, 0.4)	0.20
**Antibiotics**	Yes	3	4.0 ± 0.6	0.5 (-0.3, 1.3)	0.23	0.8 (-0.2, 1.7)	0.11
	No	25	3.5 ± 0.7	ref			
**PPIs**	Yes	5	3.5 ± 0.8	-0.007 (-0.7, 0.7)	0.98	-0.6 (-1.4, 0.3)	0.19
	No	23	3.5 ± 0.7	ref			
**Laxatives**	Yes	8	3.7 ± 0.5	0.2 (-0.4, 0.8)	0.51	-0.2 (-0.9, 0.5)	0.62
	No	20	3.5 ± 0.7	ref			
**Prebiotics/Prebiotics**	Yes	8	3.4 ± 0.8	-0.2 (-0.8, 0.4)	0.54	0.0 (-0.7, 0.6)	0.90
	No	20	3.6 ± 0.6	ref			
**Supplement**	Yes	4	3.4 ± 1.0	-0.1 (-0.9, 0.6)	0.70		
	No	24	3.6 ± 0.6	ref			
**Lung/pleural metastasis**	Yes	17	3.5 ± 0.6	-0.2 (-0.7, 0.4)	0.54		
	No	11	3.6 ± 0.7	ref			
**Brain metastasis**	Yes	5	3.3 ± 0.8	-0.3 (-1.0, 0.4)	0.36		
	No	23	3.6 ± 0.6	ref			
**Albumin**	< 3.2 g/dL	12	3.4 ± 0.6	ref			
	≥ 3.2 g/dL	16	3.6 ± 0.7	0.2 (-0.4, 0.7)	0.50		
**Cohort**	*EGFR*-WT	13	3.3 ± 0.7	ref			
	*EGFR-*mutant	15	3.8 ± 0.6	0.5 (0.1, 1.0)	0.03	0.6 (0.0, 1.2)	0.05

*BMI* Body mass index, *PPI* Proton pump inhibitor, *EGFR* Epidermal growth factor receptor, *WT* Wild-type

We also explored the potential of the Shannon index to predict treatment responses, but no association was identified in univariate or multivariate logistic regression analysis.

At the time of disease evaluation, we observed a non-significant increase of the Shannon index in the *EGFR*-WT cohort, whereas the index was similar between baseline and the time of disease evaluation in the *EGFR-*mutant cohort (Fig. 3). Following treatment, the difference in the Shannon index between the two cohorts had dissipated (*P* = 0.495, Supplement Fig. 4 ). Beta diversity was similar between the two cohorts at both baseline and the time of disease evaluation. We further investigated the microbiota diversity between responders and non-responders and found no significant differences in alpha or beta diversity in both cohorts.

**Fig. 4:**
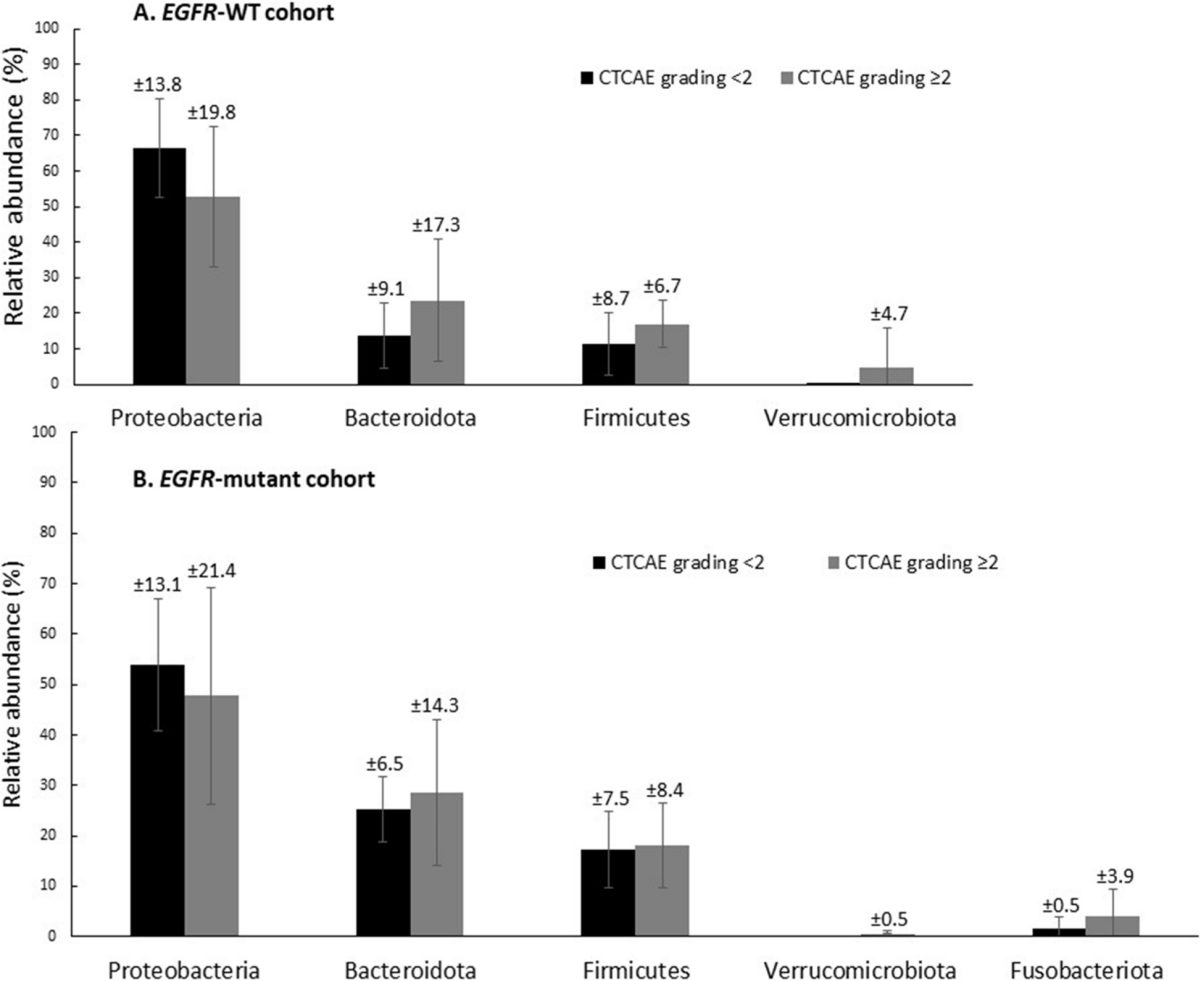
Relative abundance of gut microbiota phyla between less severe (CTCAE grading < 2) and severe adverse events (CTCAE grading ≥ 2). **A** *EGFR*-WT cohort **B** *EGFR*-mutant cohort. CTCAE: Common Terminology Criteria for Adverse Events; *EGFR*: epidermal growth factor receptor; WT: wild-type

### Association between the gastrointestinal microbiota profile and AEs

**Fig. 5:**
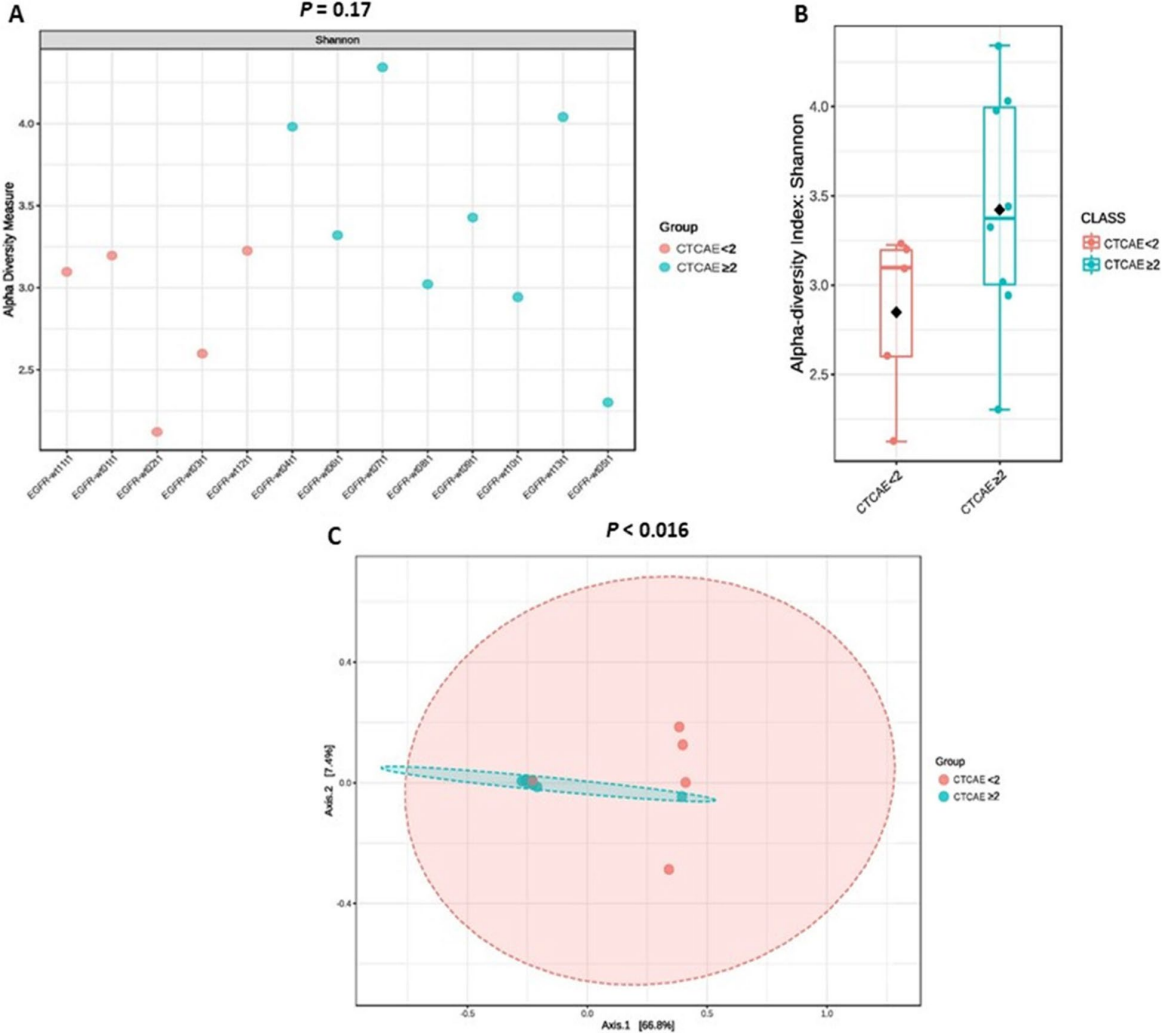
Comparison of alpha and beta diversity (Principal Coordinates Analysis plot; PCoA plot) between CTCAE grading < 2 and CTCAE grading ≥ 2 in *EGFR*-WT cohort. **A** Shannon index in each sample **B** Shannon index between CTCAE grading < 2 and CTCAE grading ≥ 2. **C**. Beta diversity (PCoA plot). CTCAE: Common Terminology Criteria for Adverse Events; EGFR-WT: epidermal growth factor receptor wild-type; PCoA plot: Principal Coordinates Analysis plot

We next examined the potential association of the microbiota composition with AEs during treatment. The microbiota profile was compared between patients with mild and severe AEs at both baseline and the time of disease evaluation. Differences in the relative abundance of microbes were observed in both groups at baseline, but the differences were more prominent in the *EGFR*-WT group. Proteobacteria were enriched in patients with mild AEs, whereas Bacteroidetes and Firmicutes were enriched in patients with severe AEs (Fig. 4). The Shannon index was numerically but not significantly higher in patients with severe AEs. We further conducted PCoA to assess beta diversity. The results revealed significant differences in microbial communities in patients with mild and severe AEs in the *EGFR*-WT cohort (Fig. 5).

**Fig. 6:**
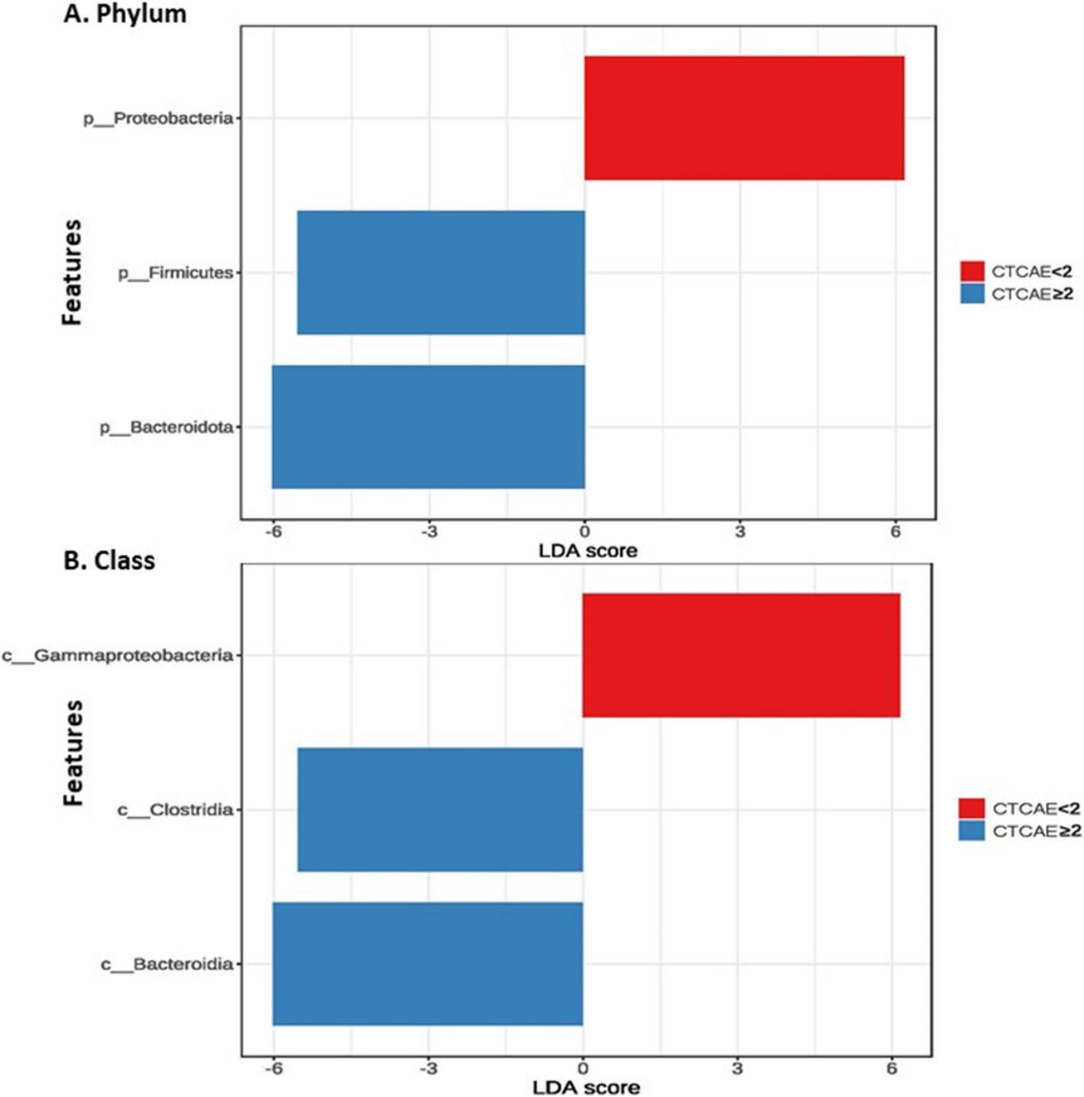
Linear discrimination analysis (LDA) identify significant microbiota between CTCAE grading < 2 and CTCAE grading ≥ 2 in *EGFR*-WT cohort A. Phylum level B. Class level. CTCAE: Common Terminology Criteria for Adverse Events; EGFR-WT: epidermal growth factor receptor wild-type; LDA: Linear discrimination analysis

We performed LEfSE analysis to identified biomarkers for AEs at the class level. In the *EGFR*-WT group, the abundance of Gammaproteobacteria was higher in patients with mild AEs (LDA score = 6), whereas those of Clostridia (LDA score =  − 5.8) and Bacteroidia (LDA score =  − 6) were higher in patients with severe AEs (Fig. 6). The fecal microbiota profile was similar between patients with mild and severe AEs in the *EGFR-*mutant cohort.

## Discussion

Our study investigated the gastrointestinal microbiota in Thai patients with advanced *EGFR*-WT (received platinum-based doublet chemotherapy) and *EGFR-*mutant NSCLC (received EGFR-TKIs). First, we explored the composition of the gastrointestinal microbiota at baseline. Generally, the gastrointestinal tract is dominated by members of four bacterial phyla: Bacteroidetes, Firmicutes, Proteobacteria, and Actinobacteria [27–29]. These data correspond with those in healthy Thai people [30, 31]. Our study found that Proteobacteria was the most abundant phylum in Thai lung cancer patients, whereas the abundance of Actinobacteria was diminished in both the *EGFR-*WT and *EGFR*-mutant cohorts. Alteration of the gastrointestinal microbiota composition has been linked to the pathogenesis of several metabolic and inflammatory diseases including cancer. Shin et al. [29] also observed a higher abundance of Proteobacteria in patients with inflammatory conditions and cancer. In mouse models, abnormal expansion of Proteobacteria is associated with dysregulation of both innate and adaptive immune responses because of interference with interleukin and CD4 + T cell function. Dysbiosis in mice increases the risks of inflammatory bowel disease and colitis-associated colorectal cancer. Moreover, previous data revealed that Firmicutes promotes colonic luminal short-chain fatty acid and modulates inflammation in mice and humans. Decreases in the abundance of Firmicutes may be associated with lung cancer development [32]. Actinobacteria, which comprises a large proportion of the healthy human microflora, has potent cancer-suppressing activity because of its metabolites [32]. Thus, the increased abundance of Proteobacteria and decreased abundance of Firmicutes or Actinobacteria at the phylum level relative to those in lung cancer patients could be emerged as a potential biomarkers of lung carcinogenesis, but we need to have the further exploration in the larger cohort, together with the Thai healthy people cohort in the future. Furthermore, we also found that the abundance of Proteobacteria was decreased and that of Bacteroidetes and Firmicutes was increased after treatment in both cohorts, although the changes were stronger in patients in the *EGFR*-WT cohort, who received chemotherapy. It has been reported that chemotherapy can alter the gastrointestinal microbiota of patients with cancer by disrupting the gastrointestinal barrier and promoting subsequent bacterial translocation [33]. Consistently, our results revealed distinctive changes in the microbiota composition after treatment compared with the findings before treatment using fecal samples. The EGFR-TKI, osimertinib, did not significantly change the relative abundance of gastrointestinal microbiota in a previous study [34], in line with our findings in the *EGFR*-mutant cohort. This result is probably attributable to the lack of a direct cytotoxic effect of EGFR-TKIs on the gastrointestinal even though one of their common AEs is diarrhea. Only three patients (20%) in our *EGFR*-mutant cohort had diarrhea (one patient with severe diarrhea), indicating a lack of microbial composition changes in the *EGFR*-mutant cohort.

Dysbiosis predisposes people to cancer by causing direct DNA damage via toxins, altering the regulation of immune and pro-inflammatory pathways, and stimulating the production of carcinogenic metabolites, dysbiosis also can potentially affect the response to cancer therapy [35]. In our study, alpha diversity at baseline significantly differed between the cohorts. A lower alpha diversity index suggests the presence of fewer species in the gastrointestinal tract in the *EGFR*-WT cohort, consistent with the differential microbiota composition between the two cohorts (Fig. 6B). Beta diversity as visualized using PCoA also revealed some differences in the microbiota between the *EGFR*-WT and *EGFR*-mutant cohorts, albeit without significance. Previous research revealed no significant reduction in the alpha diversity of the gastrointestinal in patients with lung cancer compared to healthy individuals. However, they did not show the difference between *EGFR*-WT and *EGFR*-mutant cancer, as shown in our study [34, 36]. This result is important for explaining the differences in biology between these lung cancer types.

Previous studies described a relationship between high microbiota diversity and better clinical responses to treatment with immune checkpoint inhibitors. The improved response was associated with increases of memory CD8 + T cell and natural killer cell counts in patients with in high microbiota diversity [12, 13]. However, there were no significant differences in alpha and beta diversity between responders and non-responders in both cohorts in our study, probably because of the small numbers of patients in each group.

The most common AEs in the *EGFR*-WT cohort were anemia (92.3%) and vomiting (25.0%). Interestingly, we observed a high abundance of Clostridia and Bacteroidia at baseline in patients who experienced severe AEs in the *EGFR*-WT cohort. Previous studies found Bacteroidia and Clostridia species as potentially potential enteropathogenic bacteria usually associated with diarrhea and other gastrointestinal toxicities [37, 38]. Thus, these two classes of bacteria are likely associated with the treatment toxicity, and may be used as biomarkers of chemotherapy-induced toxicity, although further research is needed.

In addition, our study explored the clinical factors associated with the response to treatment. Interestingly, we found that serum albumin level, which reflects the nutritional status of patients, affected the outcome of treatment. Patients with serum albumin level ≥ 3.2 g/dL had significantly higher treatment response rates than those with low albumin. This was consistent with previous studies identifying that the pre-treatment serum albumin level was a prognostic biomarker in cancer treatment [39]. However, most *EGFR*-mutant patients have higher serum albumin level, thus, *EGFR* status may be a confounding factor for analyzing correlation of clinical factors and response to treatment*.*

Smoking is another confounding factor in this study. 20–30% of populations have prior smoking history. Toxic chemical exposure such as nicotine, aldehydes, polycyclic aromatic hydrocarbons result in intestinal irritation and impaired mucosal immune responses. Proteobacteria increase in number with high number of pack-years of cigarette smoking in systematic review [40].

One limitation of this study was its small sample size, which might explain the findings concerning the associations of the gastrointestinal microbiota with treatment responses and AEs. More participants are needed to confirm whether the gastrointestinal microbiota can predict treatment response or toxicity.

In conclusion, this is the first study of the gastrointestinal microbiota in patients with *EGFR-WT* and *EGFR*-mutant lung cancer. Our study indicated that lung cancer disease might affect the gastrointestinal microbiota at baseline in composition and diversity. Proteobacteria was dominant in our cohort, and this phylum maybe associate with lung cancer carcinogenesis. Chemotherapy altered the gastrointestinal microbiota, whereas *EGFR*-TKIs had less effects. The findings of our pilot study highlight the potential predictive utility of the gastrointestinal microbiota for lung cancer carcinogenesis, particularly concerning cancer prevention and treatment. Studies with larger cohorts and comparison with the healthy Thai population are ongoing to confirm and further explore the gastrointestinal microbiota and its association with lung cancer.

## Supplementary Information

**Additional file 1: Supplemental Table 1.** Response of treatment. **Supplemental Figure 1.** Consort Diagram. **Supplemental Figure 2.** Comparison of relative abundance of gut microbiota phyla between responder (R) and non-responder (NR) A. EGFR-WT cohort B. EGFR-mutant cohort. **Supplemental Figure 3.** Bar chart of Phylogenetic composition of each patient according to response of treatment A. EGFR-WT cohort B. EGFR-mutant cohort. **Supplemental Figure 4.** Comparison of alpha diversity in responders (R) and non-responders (NR) in both cohorts A. EGFR-WT cohort B. EGFR-mutant cohort

## Data Availability

The datasets generated during and/or analysed during the current study are available from the corresponding author on reasonable request.
