# Supplementary material for: Gastrointestinal microbiota profile and clinical correlations in advanced EGFR-WT and EGFR-mutant non-small cell lung cancer
Source: BMC Cancer. 2022 Sep 8;22:963. doi: 10.1186/s12885-022-10050-3 (PMC9454126; doi:10.1186/s12885-022-10050-3)
Supplement: Supplementary file 1 — Additional file 1: Supplemental Table 1. Response of treatment. Supplemental Figure 1. Consort Diagram. Supplemental Figure 2. Comparison of relative abundance of gut microbiota phyla between responder (R) and non-responder (NR) A. EGFR-WT cohort B. EGFR-mutant cohort. Supplemental Figure 3. Bar chart of Phylogenetic composition of each patient according to response of treatment A. EGFR-WT cohort B. EGFR-mutant cohort. Supplemental Figure 4. Comparison of alpha diversity in responders (R) and non-responders (NR) in both cohorts A. EGFR-WT cohort B. EGFR-mutant cohort [file 12885_2022_10050_MOESM1_ESM.docx]

**Supplemental Table and Figure Legends**

Supplemental Table 1: Response of treatment

Supplemental Figure 1: Consort Diagram

Supplemental Figure 2: Comparison of relative abundance of gut microbiota phyla between responder (R) and non-responder (NR) A. *EGFR*-WT cohort B. *EGFR*-mutant cohort

Supplemental Figure 3: Bar chart of Phylogenetic composition of each patient according to response of treatment A. *EGFR*-WT cohort B. *EGFR*-mutant cohort

Supplemental Figure 4: Comparison of alpha diversity in responders (R) and non-responders (NR) in both cohorts A. *EGFR*-WT cohort B. *EGFR*-mutant cohort

**Supplemental Table 1: Response of treatment**

| **Best response, No. (%)** | **Total population**  **(N = 28)** | ***EGFR*-WT cohort**  **(N = 13)** | ***EGFR*-mutant cohort**  **(N = 15)** |
| --- | --- | --- | --- |
| **Complete response (CR)** | 0 (0) | 0 (0) | 0 (0) |
| **Partial response (PR)** | 11 (39.2) | 1 (7.6) | 10 (66.6) |
| **Stable disease (SD)** | 9 (32.1) | 5 (38.4) | 4 (26.6) |
| **Progressive disease (PD)** | 8 (28.57) | 7 (53.8) | 1 (6.6) |
| **Overall response rate (ORR)** | 11 (39.2) | 1 (7.6) | 10 (66.6) |
| **Disease control rate (DCR)** | 20 (71.4) | 6 (46.1) | 14 (93.3) |

**ORR = CR + PR; DCR = CR + PR + SD*

*EGFR*: epidermal growth factor receptor; WT: wild-type

**Supplemental Figure 1: Consort Diagram**


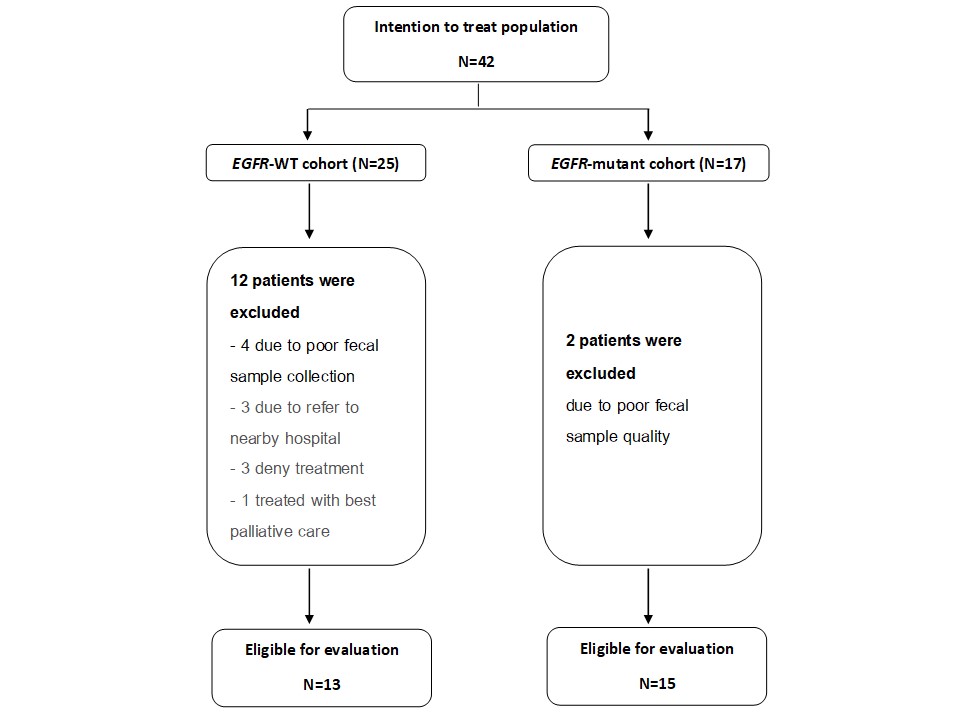


EGFR: epidermal growth factor receptor; WT: wild-type

**Supplemental Figure 2: Comparison of r**elative abundance of gut microbiota phyla between responder (R) and non-responder (NR) A. *EGFR*-WT cohort B. *EGFR*-mutant cohort


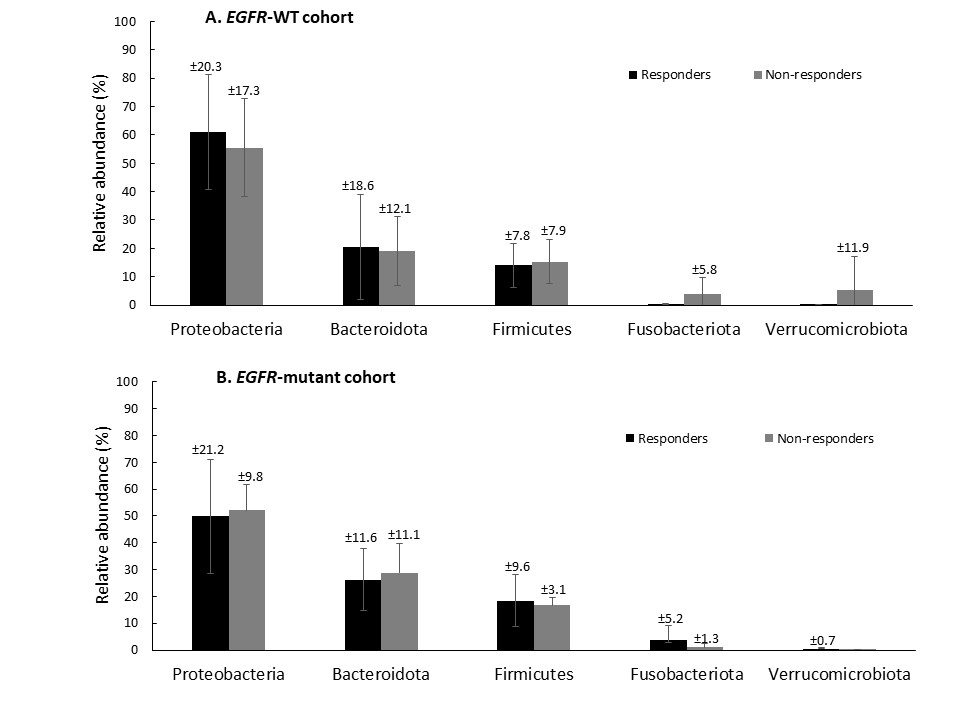


EGFR: epidermal growth factor receptor; WT: wild-type

**Supplemental Figure 3: Bar chart of Phylogenetic composition of each patient according to response of treatment A. *EGFR*-WT cohort B. *EGFR*-mutant cohort**


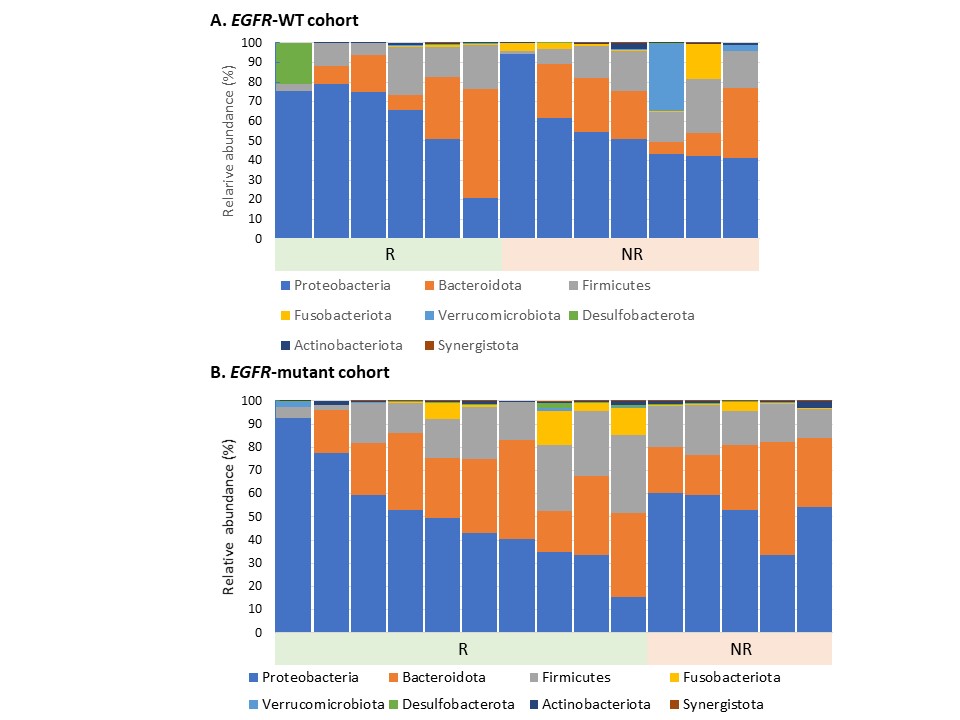


EGFR: epidermal growth factor receptor; NR: non-responders; R: responders; WT: wild-type

**Supplemental Figure 4: Comparison of alpha diversity in responders (R) and non-responders (NR) in both cohorts A. *EGFR*-WT cohort B. *EGFR*-mutant cohort**

**
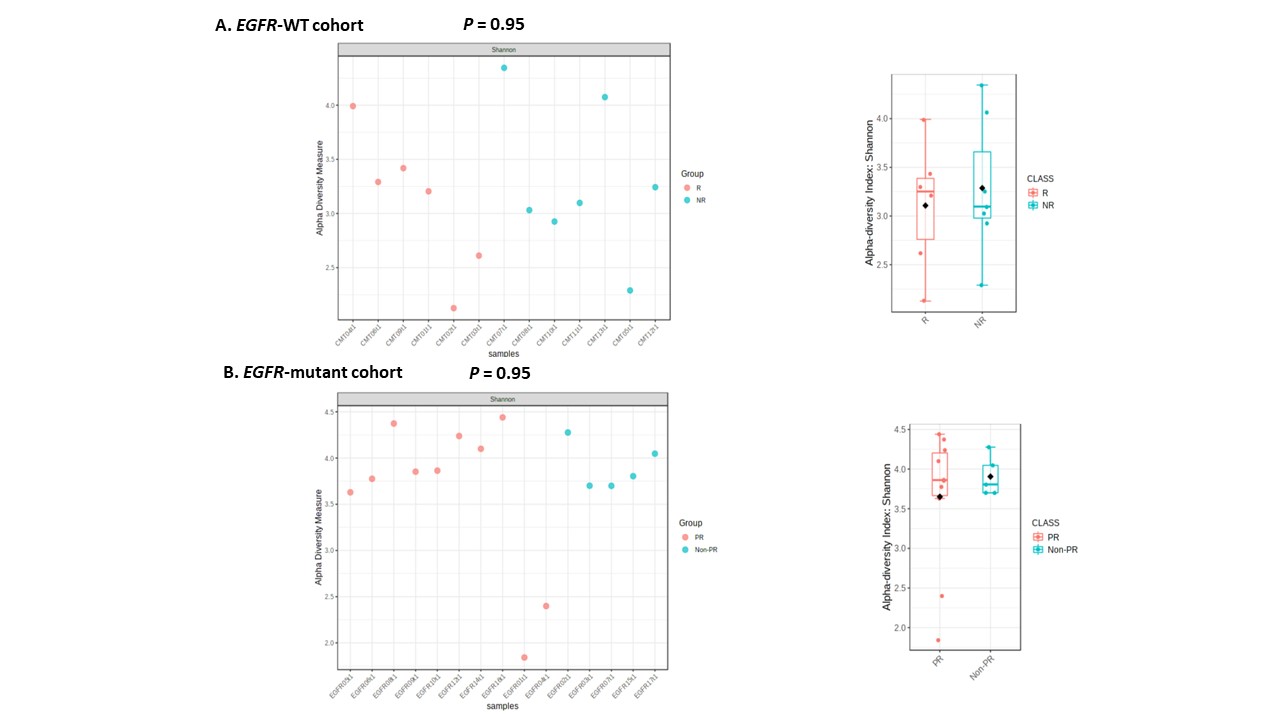
**

EGFR: epidermal growth factor receptor; NR: non-responders; R: responders; WT: wild-type
